# Supplementary figures and images for: Heterogenous Nuclear Ribonucleoprotein H1 Promotes Colorectal Cancer Progression through the Stabilization of mRNA of Sphingosine-1-Phosphate Lyase 1
Source: Int J Mol Sci. 2020 Jun 25;21(12):4514. doi: 10.3390/ijms21124514 (PMC7350029; doi:10.3390/ijms21124514)

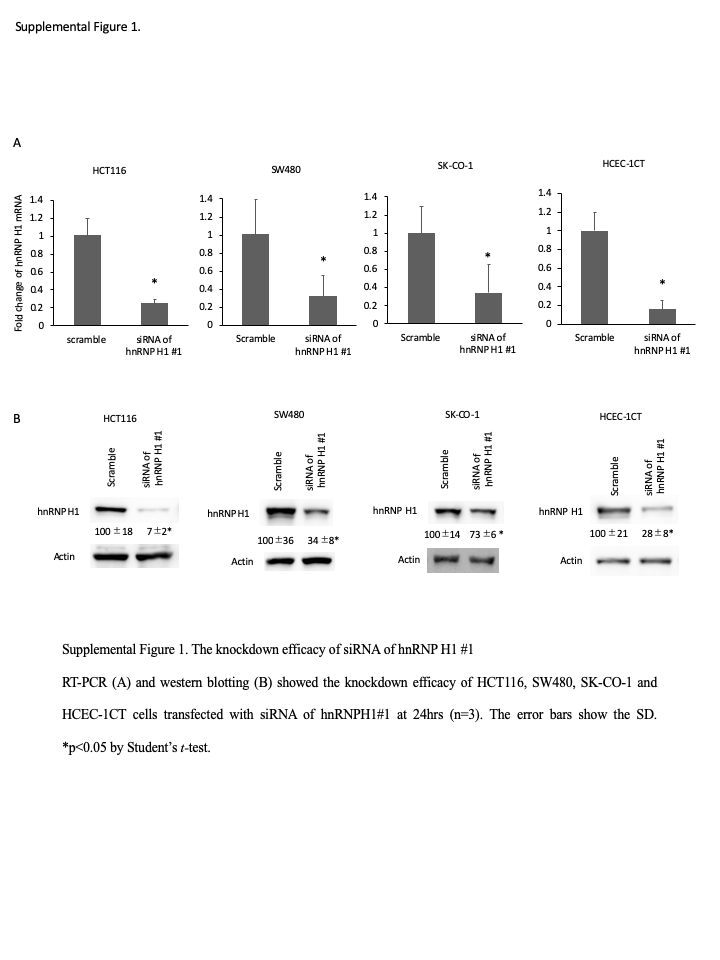

Supplement: Supplementary file 1 [file ijms-21-04514-s001.zip › supplementary files/Supplementary Figure1.tiff]

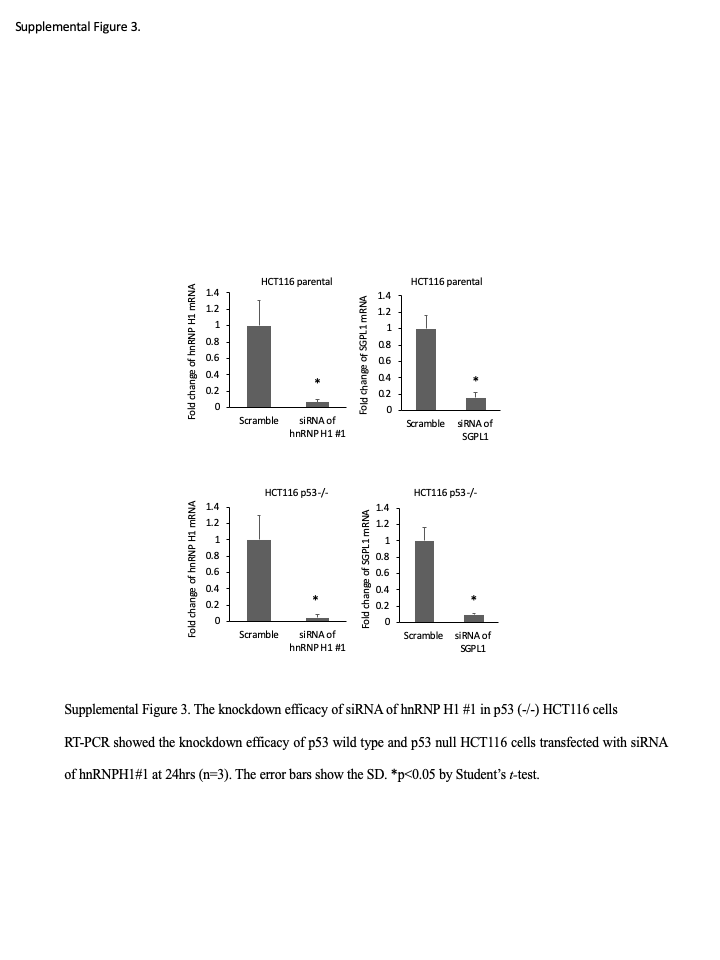

Supplement: Supplementary file 1 [file ijms-21-04514-s001.zip › supplementary files/Supplementary Figure3.tiff]

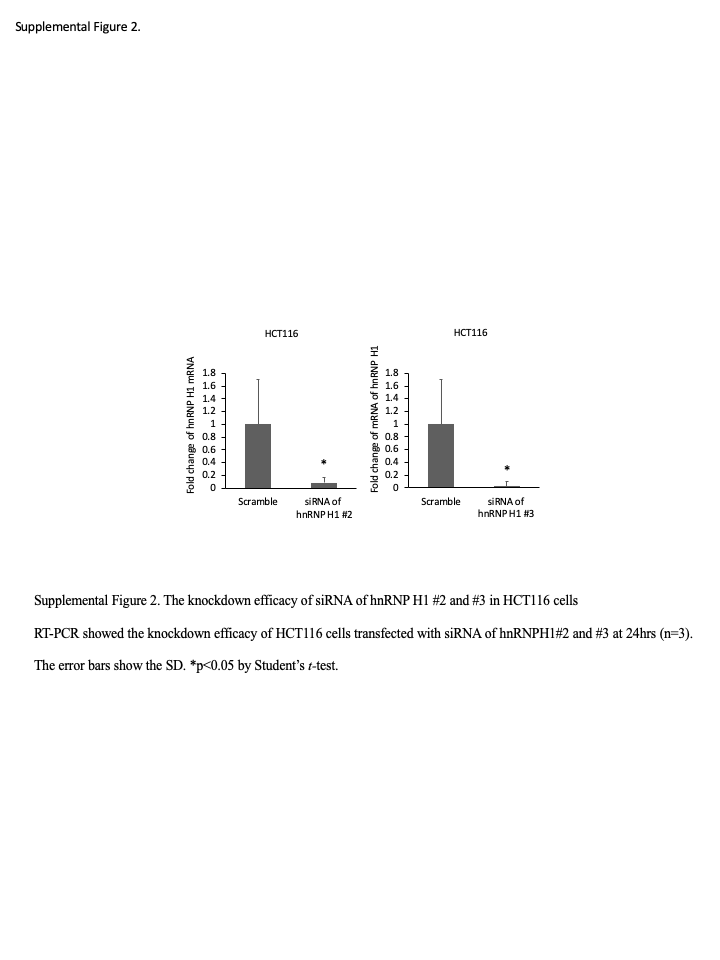

Supplement: Supplementary file 1 [file ijms-21-04514-s001.zip › supplementary files/Supplementary Figure2.tiff]
